# Comparison of the effects of an ERAS program and a single-port laparoscopic surgery on postoperative outcomes of colon cancer patients

**DOI:** 10.1038/s41598-019-48526-1

**Published:** 2019-08-19

**Authors:** Min Ki Kim, Jun-Gi Kim, Gyeora Lee, Daeyoun David Won, Yoon Suk Lee, Bong-Hyeon Kye, Jihoon Kim, In Kyu Lee

**Affiliations:** 1 Department of Surgery, Myongji Hospital, Goyang, Republic of Korea; 2 Department of Surgery, College of Medicine, The Catholic University of Korea, Seoul, Republic of Korea; 3 Department of Surgery, Incheon St. Mary’s Hospital, College of Medicine, The Catholic University of Korea, Seoul, Republic of Korea; 4 Department of Surgery, Seoul St. Mary’s Hospital, College of Medicine, The Catholic University of Korea, Seoul, Republic of Korea

**Keywords:** Surgical oncology, Colon cancer

## Abstract

Advancement of the surgical modality and perioperative care are the two main dimensions for the modern improvement of surgical outcome. The purpose of this study was to compare the effectiveness of the two by using the data from the single-port laparoscopic surgery and the early recovery after surgery (ERAS) program. Patients who underwent elective surgery for primary adenocarcinoma of the colon were divided into three groups and compared: ERAS (multi-port laparoscopic surgery with ERAS perioperative care), Conventional-SILS (single-port surgery with conventional perioperative care), or Conventional-Multi (multi-port laparoscopic surgery with conventional perioperative care). Ninety-one, 83, and 96 patients were registered, respectively. There were no differences among the three groups in baseline characteristics except pathological stage and operation site in colon. Although the ERAS group started a soft diet earlier and had earlier discharge, there were no differences in intra- and post-operative morbidity rate, readmission rate, or reoperation rate. The ERAS perioperative care was a significant factor for reducing length of hospital stay in the multivariate analysis, while single-port surgery was not. In modern laparoscopic colon cancer treatment, a systemic approach such as the ERAS program appears to be more effective than a technical approach for significantly improving short-term surgical outcomes.

## Introduction

Short- and long-term outcomes of colorectal cancer surgery are improving due to a number of advancements, including minimally invasive surgery, the principle of total mesorectal excision/complete mesocolic excision, a multidisciplinary team approach, and the development of chemotherapy in the modern era. The ERAS (Early Recovery After Surgery) program is also known to improve postoperative outcomes following colorectal surgery. ERAS aims to quickly restore patients to preoperative condition after surgery through various efforts such as minimizing fasting time in the perioperative period, encouraging exercise, and intensive control of pain^1^. ERAS is known to reduce postoperative complications, hasten recovery, and reduce the length of hospital stay^2,3^. Another approach, single-port laparoscopic surgery, is reported to not only have a cosmetic effect, but also decrease intraoperative blood loss and postoperative length of hospital stay by enhancing recovery of bowel movements due to further shortening of the incision length compared to conventional multi-port laparoscopic surgery^4^.

Both methods can improve short-term performance in colon cancer surgery, but one is a systemic approach requiring cooperation from experts in different fields, and the other is a technical approach, dependent on the ability of the surgeon. Neither has been sufficiently validated with clinical data in Korea. Currently, multi-port laparoscopic surgery with conventional perioperative care is the main surgical treatment for colon cancer in Korea^5^. To further improve postoperative surgical outcome, research is needed to determine whether the technical or systemic approach is more effective.

Hence, the purpose of this study was to compare the efficacy of ERAS, which strengthens the systemic approach, and single-port laparoscopic surgery, which strengthens the technical approach, for improving postoperative outcome.

## Methods

### Data sources and definitions

After a three-month trial period, our institution established a protocol according to the principles of ERAS for perioperative care of patients undergoing any type of colorectal resection (conventional open, multi-port laparoscopic, single-port laparoscopic, or robot-assisted laparoscopic) for primary adenocarcinoma. The protocol was registered as a critical pathway, and the clinical data of patients registered in the critical pathway were recorded prospectively. Patients registered in this data set between January and December 2017 who met study inclusion criteria (Table 1) were selected and defined as the ERAS group. The common features of this group were that the patients received multi-port laparoscopic surgery and perioperative care according to the ERAS protocol.

**Table 1:** Characteristics of study data. Data for the final analysis were extracted by adopting a narrower inclusion criteria (expressed in bold font) and all exclusion criteria from the two data sets.

	ERAS	Conventional	
		Conventional-SILS	Conventional-Multi
Operative modality	Multi-port laparoscopy	Single-port laparoscopy	Multi-port laparoscopy
Perioperative care	per ERAS protocol	per conventional care protocol (different according to individual surgeon)	
Hospital	Seoul St. Mary’s	Seoul & Incheon Mary’s	
Operative period	2017. 1–12	2011. 8–2017. 2	
Inclusion criteria	Colorectal adenocarcinoma	Colon adenocarcinoma	
	Curative resection		
	Elective surgery		
	Age 25~85	Age 18~	
Exclusion criteria	ASA grade ≥ 3		
	Emergency operation		
	Mid T ~ D colon cancer	Infectious state at admission	
	Bowel perforation		
	Bowel obstruction including stent insertion case		
	Pregnancy		
	Tumor associated with FAP, HNPCC, or IBD		
	Stage IV		
	cT4b		
	synchronous colorectal cancer		
	Other malignancy within the last 5 years		

*ERAS* early recovery after surgery, *SILS* single-incision laparoscopic surgery, *ASA* American Society of Anesthesiologists, *FAP* familial adenomatous polyposis, *HNPCC* hereditary non polypoid colorectal cancer, *IBD* inflammatory bowel disease.

The data for patients receiving conventional perioperative care was extracted from the SIMPLE (multicenter, randomized *si*ngle-port versus *multi-port l*aparoscopic surg*e*ry) trial (ClinicalTrials.gov Identifier: NCT01480128) at Seoul St. Mary’s Hospital and Incheon St. Mary’s Hospital from patients who received surgery between August 2011 and February 2017. The SIMPLE trial was a multicenter, randomized, controlled trial involving seven hospitals in Korea designed to test the hypothesis that the short-term outcome of single-port laparoscopic surgery was non-inferior to that of multi-port laparoscopic surgery in colon cancer^6^. Accordingly, patients were randomized to receive single-port or multi-port surgery, and all received conventional perioperative care. The former was defined as the Conventional–SILS (Single Incision Laparoscopic Surgery) group, and the latter as the Conventional-Multi group. In the present study, conventional perioperative care refers to perioperative care determined by the experience and knowledge of the operating surgeon according to patient and situation.

We compared all three groups (ERAS, Conventional-SILS, Conventional-Multi) as divided by surgical method (multi-port laparoscopic surgery vs. single-port laparoscopic surgery) and perioperative care fashion (conventional vs. ERAS protocol). This approach made it possible to determine whether the difference between ERAS and SILS was from the surgical method or the perioperative management approach, which would not have been possible if the comparison was only made between ERAS (multi-port laparoscopic surgery plus ERAS perioperative care) and SILS (single-port laparoscopic surgery plus conventional perioperative care) patients. The addition of the Conventional-Multi group as a control allowed us to focus on how ERAS and SILS changed short-term outcome after surgery, respectively, compared to conventional treatment. This retrospective review of prospectively collected data was approved by the institutional review board of The Catholic Medical Center, The Catholic University of Korea (study number: XC18REDI0046), and was performed in accordance with relevant guidelines and regulations. Informed consent was obtained from all participants and/or their legal guardians before surgery about the SIMPLE study or the ERAS perioperative management, respectively. Informed consent for this specific study is not required because of its retrospective design, according to the policy of our institutional review board.

Because the data were extracted from two different data sets (SIMPLE and ERAS data), we adopted a narrower range for inclusion criteria and all exclusion criteria from the two data sets. This process made the basic conditions of patients from the different data sets as homogeneous as possible. The conditions and inclusion/exclusion criteria for each group are as shown in Table 1.

### Surgeons

A surgeon from Incheon St. Mary’s Hospital who participated in the SIMPLE trial transferred to Seoul St. Mary’s Hospital in March 2017, and contributed to the data from the ERAS group. Two researchers who had been at Seoul St. Mary’s Hospital participated in surgeries for the SIMPLE trial (Conventional-Multi, Conventional-SILS) and the ERAS group, only in different time periods. All researchers who participated in the SIMPLE trial from the beginning of the study were required to have conducted more than 50 single-port surgeries for colon cancer. Those joining the study later were verified by other researchers through an unedited single-port surgery video for colon cancer^6^. Consequently, three verified surgeons participated in the surgeries of each group defined in this study.

### ERAS protocol

ERAS prescribes multiple items for each period before, during, and after surgery, and these items have target values. The percentage of all items that are executed is defined as compliance, and a higher compliance rate is correlated with stronger effects from ERAS^7^. The components of the ERAS protocol of Seoul St. Mary’s Hospital are outlined in Supplementary Table 1 and were selected in consideration of Korean norms based on several known ERAS-related references^1,8^. The discharge criteria for ERAS patients were (1) no evidence of complications, (2) tolerance of a soft diet (SD), (3) walking without assistance, and (4) well-controlled pain on oral medicine.

### Endpoint

We presumed that there was no difference in incidence of complications via ERAS and SILS based on the interim analysis of the SIMPLE study^6^ and outcome data obtained from the initial three-month trial period of ERAS. Accordingly, we hypothesized that the postoperative length of hospital stay would reflect differences between the two approaches and regarded it as the primary endpoint. Secondary endpoints were complication rate, timing of diet resumption, pain score as assessed by visual analogue scale (VAS) on days 1, 2, and 3 after surgery, change in inflammatory state following surgery, readmission rate, and re-operation rate. Severity of complications was based on the Clavien-Dindo classification^9^. In the SIMPLE trial, the definition of postoperative ileus was “failure of gas passing combined with radiological evidence of bowel dilatation until postoperative day 3 and causing delay of oral intake.” Since the ERAS group resumed diet regardless of gas out status, we separately re-defined ileus in the present study as “a failure or difficulty of gas passing combined with radiological evidence of bowel dilatation that caused interruption of a meal,” and medical records were reviewed and compared according to this definition.

Changes in serum white blood cell (WBC) level and neutrophil lymphocyte ratio (NLR) during the perioperative period (on the day before surgery, immediately after surgery, and postoperative day 1) were compared among the groups to determine if there was a difference in the degree of inflammatory reactions induced by surgical stress. Serum WBC count below 10,000 × 10^3^/mL was defined as normal, and NLR below 5 as normal.

### Statistical analysis

Continuous variables were expressed as median (range), and the differences among three groups were compared with the Kruskal-Wallis test. If the differences were significant, a Mann Whitney U test was performed for inter-group analysis. Categorical variables were presented as number (%) and compared using Chi-square or Fisher’s exact test. Multiple regression analysis were performed to evaluate the true effect of ERAS perioperative care and single-incision laparoscopic surgery on the postoperative length of hospital stay. Variables for perioperative care (Non-ERAS vs. ERAS) and operative method (multiport laparoscopic vs. single-incision laparoscopic surgery) were newly coded for these multivariate analysis. Confounding factors were adjusted for in these analysis included the following: age, sex, body mass index (BMI), name of operation, history of previous abdominal surgery, American Society of Anesthesiologists (ASA) grade, preoperative serum carcinoembryonic antigen (CEA) level, operation time, estimated blood loss, intraoperative complication, postoperative complication (only for analyzing about the effect on postoperative length of stay), concomitant other abdominal organ resection, and pathologic stage. All analysis was performed with IBM SPSS ver. 24 (IBM Co., Armonk, NY, USA), and statistical significance was defined as a p-value < 0.05 (for the inter-group analysis after significant Kruskal-Wallis test results, a p-value < 0.017 was defined as statistically significant according to Bonferroni’s method).

## Results

A total of 91 patients in the ERAS group, 83 patients in the Conventional-SILS group, and 96 patients in the Conventional-Multi group were eligible based on our inclusion and exclusion criteria. In the ERAS group, the protocol compliance rate was 74.9%. Age, sex, BMI, history of previous abdominal surgery, and preoperative serum CEA level did not differ among the three groups. However, the ERAS group had significantly fewer pathologic stage 0 patients (ERAS vs. Conventional-SILS vs. Conventional-Multi; 1.1% vs. 14.5% vs. 6.3%, p = 0.001) and more stage III patients compared to the other two groups (41.8% vs. 21.7% vs. 36.5%, p = 0.001) (Table 2).

**Table 2:** Baseline patient characteristics.

		ERAS (n = 91)	Conventional-SILS (n = 83)	Conventional -Multi (n = 96)	p-value
Age	years	64.5 (31–84)	61 (34–84)	61.5 (38–81)	0.114
Sex	male	42 (46.2%)	48 (57.8%)	56 (58.3%)	0.183
BMI	kg/m^2^	23.5 (15.7–38.4)	24.1 (17.8–30.5)	24.3 (18.0–34.1)	0.490
ASA	1	23 (25.3%)	39 (47.0%)	52 (54.2%)	<0.001
	2	68 (74.7%)	44 (53.0%)	44 (45.8%)	
History of abdominal surgery	yes	16 (17.6%)	13 (15.7%)	28 (29.2%)	0.058
preoperative CEA level	mg/dL	2.13 (0.50–66.41)	2.03 (0.50–133.0)	1.80 (0.50–25.6)	0.982
Stage	0	1 (1.1%)	12 (14.5%)	6 (6.3%)	0.001
	1	28 (30.8%)	19 (22.9%)	29 (30.2%)	
	2	24 (26.4%)	34 (41.0%)	26 (27.1%)	
	3	38 (41.8%)	18 (21.7%)	35 (36.5%)	

*ERAS* early recovery after surgery, *SILS* single-incision laparoscopic surgery, *BMI* body mass index, *AS*A American Society of Anesthesiologists, *CEA* carcinoembryonic antigen.

In the ERAS group, a significant number of patients had their lesion in the cecum ~ proximal T colon, and right hemi-colectomy (RHC) or extended RHC were significantly higher than in the other two groups (49.5% vs. 22.9% vs. 22.9%, p < 0.001). There were no differences in incidence of other concomitant abdominal organ resection, operation time, or intraoperative complications. Estimated blood loss (EBL) during surgery was higher in the ERAS group, but the difference was not clinically meaningful (50 [10–500] vs. 30 [0–800] vs. 20 [0–300], p < 0.001) (Table 3).

**Table 3:** Surgical information.

		ERAS (n = 91)	Conventional-SILS (n = 83)	Conventional-Multi (n = 96)	p-value
Operation name	RHC	45 (49.5%)	19 (22.9%)	22 (22.9%)	<0.001
	AR	46 (50.5%)	64 (77.1%)	74 (77.1%)	
Co-operation	Yes	3 (3.3%)	1 (1.2%)	0 (0.0%)	0.162
Operation time	min	169.5 (70–315)	140 (64–496)	135 (61–420)	0.266
EBL	mL	50 (10–500)	30 (0–800)	20 (0–300)	<0.001
Intraoperative complication	Yes	2 (2.2%)	8 (9.6%)	3 (3.1%)	0.065

*ERAS* early recovery after surgery, *SILS* single-incision laparoscopic surgery, *RHC* right hemicolectomy, *AR* anterior resection, *EBL* estimated blood loss.

The onset of soft diet (SD) was three days earlier in the ERAS group than in the other two groups (1[1–12] vs. 4[2–9] vs. 4[2–14], p < 0.001), and the postoperative hospital stay was one day shorter (5 [3–29] vs. 6 [4–13] vs. 6 [5–26], p < 0.001). There were no significant differences in postoperative complications, Clavien-Dindo classification grade 3 or higher complication rates, readmission rates, and reoperation rates among the three groups (Table 4). Details of complications are shown in Supplementary Table 2.

**Table 4:** Postoperative outcomes.

		ERAS (n = 91)	Conventional-SILS (n = 83)	Conventional-Multi (n = 96)	p-value
Resumption of soft diet	day	1 (1–12)	4 (2–9)	4 (2–14)	<0.001
Postoperative LOS	day	5 (3–29)	6 (4–13)	6 (5–26)	<0.001
Postoperative complication	Yes	7 (7.7%)	3 (3.6%)	5 (5.2%)	0.534
Postoperative complication grade	≥IIIa	1 (1.1%)	1 (1.2%)	2 (2.1%)	1.000
Reoperation	Yes	1 (1.1%)	1 (1.2%)	2 (2.1%)	1.000
Readmission	Yes	1 (1.1%)	1 (1.2%)	1 (1.0%)	1.000

*ERAS* early recovery after surgery, *SILS* single-incision laparoscopic surgery, *LOS* length of stay. Postoperative complication was classified according to the Clavien-Dindo classification.

Multiple regression analysis was used to test if the perioperative care or operative method have significant effect on the length of stay (R^2^ = 0.444, Adjusted R^2^ = 0.406, F = 11.788, p < 0.001). The ERAS perioperative care was a significant independent factor for reducing postoperative length of hospital stay (β = −0.326, p < 0.001) regardless of operation site or stage. The most significant factor for the length of stay was postoperative complications. The operation method, or whether it was performed by single-incision or multiport laparoscopic surgery, showed no effect on the postoperative length of stay (β = −0.042, p = 0.462) (Table 5).

**Table 5:** Multiple regression analysis for evaluating significant factors on postoperative length of hospital stay.

		Unstandardized Coefficients		Standardized Coefficients	t	Sig.
		B	Std. Error	Beta		
(Constant)		4.372	1.368		3.197	0.002
Perioperative care	ERAS	−1.674	0.304	−0.326	−5.512	<0.001
Operation method	SILS	−0.221	0.300	−0.042	−0.736	0.462
Age	years	0.009	0.012	0.040	0.721	0.472
Sex	female	0.031	0.248	0.006	0.126	0.900
BMI	kg/m^2^	0.044	0.037	0.057	1.163	0.246
Operation name	AR to RHC	0.285	0.270	0.055	1.053	0.293
History of previous abdominal surgery	yes	0.212	0.302	0.036	0.700	0.485
ASA	grade II (to I)	0.072	0.277	0.015	0.259	0.796
Preoperative serum CEA level	mg/dl	0.002	0.011	0.008	0.169	0.866
Operation time	minutes	0.001	0.002	0.033	0.583	0.560
Estimated blood loss	ml	0.001	0.001	0.041	0.700	0.485
Intraoperative complication	yes	−0.262	0.601	−0.023	−0.437	0.663
postoperative complication	yes	6.155	0.510	0.584	12.071	<0.001
concomitant other abdominal organ resection	yes	−0.037	0.972	−0.002	−0.038	0.970
Stage = 0		1.027	0.493	0.109	2.082	0.038
Stage = 1		0.213	0.308	0.039	0.692	0.489
Stage = 3		−0.134	0.296	−0.026	−0.454	0.651

*ERAS* early recovery after surgery, *SILS* single-incision laparoscopic surgery, *BMI* body mass index, *AR* anterior resection, *RHC* right hemicolectomy, *ASA* American Society of Anesthesiologists, *CEA* carcinoembryonic antigen.

**Figure 1:**
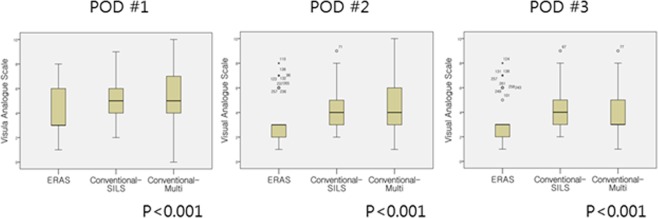
Box plot graphs of postoperative pain score presented with visual analogue scale (VAS). Postoperative pain scores were significantly lower at postoperative day 1, 2, and 3 in the ERAS group than in the other two groups. POD, Post-Operative Day (The number of outliers of the ERAS, Conventioal-SILS and Conventional-Multi group was as follows; POD 1: 0. 0. 0, POD 2: 9, 1, 0, POD 3: 8, 1, 1. However, they were included in the final analysis because a nonparametric method, of which results are robust to the presence of outliers, was used to compare the VAS levels among the groups).

Postoperative VAS scores were lower at postoperative day 1 (3 [1–8] vs. 5 [2–9] vs. 5 [0–10], p < 0.001), day 2 (3 [1–8] vs. 4 [2–9] vs. 4 [1–10], p < 0.001), and day 3 (3 [1–8] vs. 4 [2–9] vs. 3 [1–9], p < 0.001) in the ERAS group than in the other two groups (Fig. 1). In the subgroup analysis, the Conventional-SILS group had no clinical differences compared to the Conventional-Multi group.

However, the analysis of inflammatory conditions represented by serum WBC level and NLR did not identify any significant differences among the groups. Preoperatively, there was no difference in the proportion of patients with abnormal WBC level among the three groups (ERAS vs. Conventional-SILS vs. Conventional-Multi; 4.4% vs. 6.0% vs. 5.2%, p = 0.941), and the ERAS group was relatively small in terms of the proportion of patients with elevated WBC level after surgery (60.4% vs. 73.5% vs. 72.9%, p = 0.070, Table 6). A similar tendency was seen when comparison was made with only patients showing preoperative normal WBC levels (58.6% vs. 74.1% vs. 73.4%, p = 0.051). However, on postoperative day 1, the proportion of patients with elevated WBC was the highest in the ERAS group, thus there was no constant tendency by group postoperatively. Furthermore, there was no difference among the three groups in the proportion of patients with elevated NLRs after surgery among those with preoperative normal NLRs (89.7% vs. 79.7% vs. 80.7%, p = 0.149).

**Table 6:** Percentage of patients with a serum white blood cell level 10000 × 10^3^/mL or higher.

	ERAS (n = 91)	Conventional-SILS (n = 83)	Conventional-Multi (n = 96)	p-value
Preoperative	4 (4.4%)	5 (6.0%)	5 (5.2%)	0.941
Postoperative Day #0	55 (60.4%)	61 (73.5%)	70 (72.9%)	0.070
Postoperative Day #1	52 (57.8%)	27 (32.5%)	36 (37.9%)	0.008

*ERAS* early recovery after surgery, *SILS* single-incision laparoscopic surgery.

## Discussion

Advancement of the surgical modality (technical approach) and perioperative care (systemic approach) are the two main dimensions for the modern improvement of surgical outcome. However, it is often difficult to compare the effectiveness of the two approach directly in clinical setting.

In this study, we found that pain score and postoperative length of hospital stay were lower in the ERAS group, which strengthened perioperative care over conventional multi-port laparoscopic surgery plus conventional perioperative care, while there was no improvement compared to the existing therapy in the SILS group, which strengthened the technical approach in the surgical treatment of colon cancer.

Early ambulation, early diet resumption, and aggressive pain control, which are important components of ERAS, have already been shown to help patients recover after colorectal cancer surgery^10^. ERAS, which is a comprehensive combination of these therapies, also reduces complications and postoperative length of hospital stay and has recently been reported to benefit long-term survival rates^7,11^. Nonetheless, ERAS is reluctantly adopted in actual practice by colorectal units, because the approach requires the surgeon to set up a multi-disciplinary team, which means that the institution must be large enough to support such a team^12,13^. In Korea, only limited experience with ERAS has been reported, and in practice, there had been no institutions that have introduced ERAS as a routine program^14,15^.

Seoul St. Mary’s Hospital identified the efficacy of ERAS based on literature review and attendance at conferences and then introduced ERAS to selected colorectal cancer patients starting in October 2016. From January 2017, critical pathways according to the ERAS principle were established for colon and rectal cancer, and the clinical data of those patients who met the inclusion criteria for critical pathways were prospectively collected. Accordingly, it should be noted that the patients included in this study were highly selective, and this should be considered a report of early experience with ERAS.

Nevertheless, as in previous studies, ERAS patients in our study showed a reduction in hospital days without any difference in complications, even with early resumption of diet. This finding is particularly interesting considering that the ERAS group had significantly more patients undergoing surgery for right-side colon cancer, which is known to be prone to postoperative ileus^16,17^. There was a difference in the Conventional-SILS and Conventional-Multi groups, in that they resumed diet only after gas passage compared to the ERAS group.

In the SIMPLE trial, the study protocol was designed to compare the rate of recovery of intestinal motility between single- and multi-port laparoscopic surgery. Therefore, it cannot be concluded that the recovery of intestinal motility was slower in the Conventional-SILS or Conventional-Multi group compared to ERAS. However, given that the day SD was resumed was not different between the Conventional-SILS and Conventional-multi groups, it is clear that the technical approach of single-port surgery alone did not lead to accelerated recovery of bowel movement over multi-port laparoscopic surgery. In other words, the technical approach alone in this laparoscopic surgery era was not responsible for patient improvement. It should be noted that the various approaches within ERAS to promote recovery of intestinal motility did not cause any problems, even when the actual diet was resumed quickly.

Although the change from conventional open surgery to laparoscopic surgery may have accelerated recovery of intestinal motility through reduction of tissue handling and reduction of EBL^18,19^, the change from multi-port surgery to single-port caused little improvement, because the intra-corporeal procedure was actually minimally different between the two modalities. This may also be true for other technical approaches, such as robotic surgery. It is difficult to achieve a bigger difference in postoperative recovery than the current multi-port laparoscopic system by changing the technical approach without moving in a different direction from laparoscopic surgery.

On the other hand, many of the organized components of ERAS are not used in actual practice, although individually they have been shown to accelerate recovery after surgery through several individual studies. The proven effects of the components of the ERAS to restore a patient to the pre-operative state as quickly as possible include early resumption of diet and early recovery and discharge, as shown in this study.

ERAS was developed when most colorectal cancer surgery was performed with a conventional open method, and the early recovery and reduction of complications that ERAS is known for were compared with conventional perioperative care mostly after open surgery. However, the effect of ERAS on reduction of complications may be offset by the advantages of laparoscopic surgery, and ERAS with laparoscopic surgery has been shown to only reduce the length of hospital stay^20,21^. Similar to other reports, we observed no difference in complications among the groups and only shorter hospitalization in the ERAS group. This means that the technical development of single-port over multi-port laparoscopy has not brought about the same impact on complications and recovery as the development from laparotomy to laparoscopic surgery.

Pain and postoperative inflammatory status were investigated to determine factors that could reduce the postoperative length of hospital stay in the ERAS group. Pain at POD # 1, 2, and 3 on the VAS scale was significantly lower in the ERAS group than in the other two groups. Epidural patient-controlled analgesia (PCA) is a representative component of ERAS, with 72.5% (n = 66) of patients in the ERAS group of our study receiving epidural anesthesia. However, according to Hubner *et al*., epidural anesthesia has no significant difference in pain control compared to PCA, but may cause hemodynamic instability, which may lead to a longer hospital stay^22^. We performed subgroup analysis between the patients who received epidural PCA and those who received intravenous PCA in the ERAS group and found no difference in pain score. Therefore, epidural anesthesia alone could not be interpreted as a key element for reduced postoperative pain in the ERAS group. The various components of ERAS supposedly comprehensively control postoperative body fluids and inflammatory state, resulting in effective pain control, which may have affected early discharge.

Retrospective review of postoperative inflammatory status in each group was based on the assumption that the inflammatory regulatory function of ERAS promoted the recovery of patients in diet, exercise, and pain, thus shortening the length of hospital stay. The proportion of patients whose serum WBC level was 10000 × 10^3^/mL or higher was similar among the groups preoperatively, but showed a tendency toward higher occurrence in the other two groups postoperatively compared to the ERAS group. The same was true about the proportion of patients whose postoperative serum WBC level was 10000 × 10^3^/mL or higher among those with a normal preoperative serum WBC level. However, there were no definite clues to the inflammatory state because serum WBC level and NLR of the ERAS group showed a higher tendency on POD # 1.

These findings suggest that ERAS may be associated with a lower inflammatory state than seen in the other two groups, but only in the immediate period after surgery. However, differences among the groups such as stage and surgical site limit the interpretation of these results. In addition, major inflammatory markers such as C-reactive protein (CRP), which are known to reflect the stress of surgery and are the main marker of complications in colorectal surgery^23^, were not measured at the same time period in each group due to the retrospective nature of this study. Further research is needed to better understand this issue. The development of technical approaches such as SILS and robotic surgery may be beneficial to individual patients, but there are not many surgeons who can perform cancer surgery with this technology^24,25^, nor can all referred hospitals provide, train, and support surgeons with this technology. Furthermore, as we found, these surgeries that represent the next technical step after multi-port laparoscopic surgery did not make any difference in postoperative patient outcome. In contrast, while maintaining a relatively widespread and easy-to-use multi-port laparoscopic system, it is relatively easier and more economic to employ professional nurses, pharmacists, nutritionists, and rehabilitation therapists as a team to provide ERAS rather than recruit or train surgeons. Therefore, while we recommend that individual surgeons work to improve their technique, the introduction of ERAS across hospital or social systems would likely have a greater effect on patients as a whole.

This study has several limitations: First, it is a small retrospective study. Therefore, selection bias cannot be excluded. However, this bias was minimized by selecting study subjects with the same eligibility criteria from the two different data sets. Second, the timing of treatment for each group of patients was different. A comparison of pre- and post-ERAS introduction in a single province in Canada concluded that it is not sure whether difference in outcome was due to time trend or ERAS^26^. However, in practice, it would be difficult to carry out a study designed to compare SILS with conventional perioperative care in one group and multi-port laparoscopic surgery with ERAS in another group. In our institution, both the single-port surgery and ERAS care were separately applied, but performed at different times, so these comparisons were possible. Third, because medical differences such as inflammation were not clearly presented, we cannot conclude whether the difference in length of postoperative hospital stay was due to differences in the medical condition of the patients or differences in the medico-social system. In patients who received ERAS, fulfillment of clear criteria – tolerance of a soft diet, able to walk without help, controlled pain with oral medication, no evidence of complications - was necessary for hospital discharge, but in the Conventional-SILS or Conventional-Multi group, discharge was decided according to the clinical judgement of the surgeon in each case. Nevertheless, the implication of this study’s results is still constant in spite of this limitation. Our results indicated that pre- and postoperative treatment guided by systemic criteria and goals could lead to shorter hospital stays compared to treatment relying on individual judgment and surgical technique.

## Conclusion

A systematic approach through ERAS resulted in early dietary resumption, shorter hospital stays, and appropriate control of postoperative pain without increases in complications or readmission rates compared to conventional perioperative care with laparoscopic colon cancer surgery. On the other hand, the technical approach of single-port surgery on its own was not difference from conventional treatments. Thus, the most effective performance improvement in current colon cancer treatment will come from spreading the introduction of ERAS perioperative care.

## Supplementary information

Sup. Table 1 & 2
