# Supplementary material for: Comparison of the effects of an ERAS program and a single-port laparoscopic surgery on postoperative outcomes of colon cancer patients
Source: Sci Rep. 2019 Aug 19;9:11998. doi: 10.1038/s41598-019-48526-1 (PMC6700146; doi:10.1038/s41598-019-48526-1)
Supplement: Supplementary file 1 — Sup. Table 1 & 2 [file 41598_2019_48526_MOESM1_ESM.docx]

**Comparison of the effects of an ERAS program and a single-port laparoscopic surgery on postoperative outcomes of colon cancer patients**

Min Ki Kim^1^, Jun-Gi Kim^2^, Gyeora Lee^3^, Daeyoun David Won^4^, Yoon Suk Lee^4^, Bong-Hyeon Kye^4^, Jihoon Kim^3^, In Kyu Lee^4^

^1^Department of Surgery, Myongji Hospital, Goyang, Republic of Korea

^2^Department of Surgery, College of Medicine, The Catholic University of Korea, Seoul, Republic of Korea

^3^Department of Surgery, Incheon St. Mary’s Hospital, College of Medicine, The Catholic University of Korea, Seoul, Republic of Korea

^4^Department of Surgery, Seoul St. Mary’s Hospital, College of Medicine, The Catholic University of Korea, Seoul, Republic of Korea

**Correspondence address: In Kyu Lee, MD, PhD**

Department of Surgery, Seoul St. Mary’s Hospital, College of Medicine, The Catholic University of Korea, 222, Banpo-daero, Seocho-gu, Seoul, 06591, Republic of Korea

Tel: 82-2-2258-6104, Fax: 82-2-595-2822, E-mail: cmcgslee@catholic.ac.kr

**Supplementary Table 1** ERAS protocol of our institution

| Period | Component | Content |
| --- | --- | --- |
| Preoperative | Preadmission patient education |  |
|  | Preoperative oral carbohydrate treatment | by 2 hours before surgery |
|  | Preoperative formula intake | parenteral nutrient solution after mechanical bowel preparation |
|  | Thrombosis prophylaxis | preoperative vascular surgery team consultation  application of a pneumatic compression pump |
|  | Antibiotics prophylaxis | administered at 30 minutes before incision |
| Intraoperative | Epidural or spinal anesthesia |  |
|  | Body temperature preservation | Use of air warmer and trans-esophageal monitoring device |
|  | Restrictive fluid strategy | crystalloid 2-4 cc/hr |
|  | PONV prophylaxis | administered before the end of surgery |
| Postoperative | Postoperative epidural analgesia | using at least one day of PCA through epidural route |
|  | Effective pain control | well controlled pain with NSAID only |
|  | Balanced fluids | daily total fluid level 500 cc or less |
|  | Stimulation of gut motility I | laxatives used |
|  | Stimulation of gut motility II | chewing gum used |
|  | Termination of urinary drainage | withdrawal of Foley catheter before the third postoperative day |
|  | Drainage remove | until the third postoperative day |
|  | Termination of intravenous fluid infusion | until the third postoperative day |
|  | Mobilization on day of surgery | postoperative movement outside the bed |
|  | Mobilization on postoperative day 1 | over 4 hours |
|  | Mobilization on postoperative day 2 | over 6 hours |
|  | Mobilization on postoperative day 3 | over 6 hours |
|  | Energy intake on day of surgery, postoperatively | intake 200 kcal or more |
|  | Energy intake on postoperative day 1 | intake 500 kcal or more |

*ERAS* early recovery after surgery, *PONV* postoperative nausea and vomiting, *PCA* patient controlled analgesia, *NSAID* non-steroidal anti-inflammatory drug.

**Supplementary Table 2** Postoperative complications

|  | ERAS | Conventional-SILS | Conventional-Multi | p value |
| --- | --- | --- | --- | --- |
| ileus | 3 | 0 | 1 | 0.180 |
| intraabdoiminal abscess | 0 | 0 | 1 (IIIb) | 0.403 |
| anastomotic leak | 2 (1-V) | 1 (IIIb) | 1 | 0.782 |
| bloody discharge from drain | 1 | 0 | 0 | 0.373 |
| dyspnea | 1 | 0 | 0 | 0.373 |
| small bowel hematoma | 0 | 0 | 1 (IIIb) | 0.403 |
| chyle ascites | 0 | 2 | 1 | 0.317 |

*ERAS* early recovery after surgery, *SILS* single incision laparoscopic surgery

Grades of complications are expressed in brackets only for Clavien-Dindo classification grade IIIa or higher.
